# Survival among medically insured and treated head and neck cancer patients with and without HIV in South Africa

**DOI:** 10.1111/hiv.70143

**Published:** 2025-11-19

**Authors:** Precious Kefilwe Motlokwa, Yann Ruffieux, Chido Chinogurei, Andreas D. Haas, Naomi Folb, Eliane Rohner

**Affiliations:** ^1^ Princess Marina Hospital Gaborone Botswana; ^2^ Faculty of Dentistry University of the Western Cape Cape Town South Africa; ^3^ Institute of Social and Preventive Medicine University of Bern Bern Switzerland; ^4^ Centre for Integrated Data and Epidemiological Research, School of Public Health University of Cape Town Cape Town South Africa; ^5^ Medscheme Cape Town South Africa; ^6^ Division of Clinical Pharmacology, Department of Medicine University of Cape Town Cape Town South Africa

**Keywords:** head and neck cancer, HIV, South Africa, survival

## Abstract

**Objectives:**

To assess the association of HIV and overall survival among medically insured head and neck cancer (HNC) patients in South Africa and to examine factors associated with the type of treatment received.

**Methods:**

We used reimbursement claims data from a South African medical insurance scheme database from 1 January 2011 to 1 July 2020. We included individuals with at least two International Classification of Diseases (ICD‐10) codes for HNC and at least one cancer treatment code within 180 days of the HNC diagnosis. We used logistic regression to identify factors associated with receiving a specific type of cancer treatment and Cox proportional hazards models to examine factors associated with all‐cause mortality.

**Results:**

We included 566 HNC patients, 49 of whom lived with HIV and 383 were male. Patients with HIV were substantially younger at HNC diagnosis (median age: 52.5 years) than patients without HIV (median age: 61.6 years). We found no clear association between HIV status and the treatment type received. The median survival was 3.78 years (95% confidence interval [CI] 3.01–6.08) and the five‐year survival was 46.0% (95% CI 41.2%–51.5%). The risk of death was higher among patients with HIV than those without HIV (adjusted hazard ratio 1.68; 95% CI 1.00–2.81).

**Conclusion:**

Medically insured HNC patients in South Africa with HIV had higher mortality than those without HIV. This underscores the importance of tailored cancer care strategies for HNC patients with HIV.

## INTRODUCTION

Head and neck cancers (HNCs) include cancers arising from the oral cavity, oropharynx, nasopharynx, hypopharynx, larynx, nasal cavity, paranasal sinuses, and salivary glands [1]. In 2020, HNCs were the seventh most common malignancy worldwide with the oral cavity being generally the most frequent cancer site [2]. However, the incidence rates of HNCs vary greatly across geographic regions [3, 4]. In sub‐Saharan Africa, HNCs were the fifth most common cancer type in 2018 [5]. Men are more often diagnosed with HNCs than women [2]. Tobacco use and alcohol consumption are important risk factors for HNCs, especially those arising from the oral cavity and larynx, while cancers arising from the oropharynx are increasingly associated with human papillomavirus (HPV) infection [6, 7, 8]. Additional risk factors for HNCs include infections with other oncogenic viruses, such as Epstein‐Barr virus (EBV), immunodeficiency, ultraviolet (UV) radiation, especially UVB, chronic inflammation and genetic factors [2]. People with HIV (PWH) have a 2‐3 fold increased risk of developing HNC [9, 10]. Apart from other risk factors, this could be attributed to HIV‐induced immunosuppression hindering the control of oncogenic viruses as well as direct effects of HIV replication [10].

Despite prevention efforts, such as tobacco and alcohol consumption cessation, tobacco taxation, and HPV vaccination, HNC incidence rates are rising globally [7, 11]. HNCs are generally treated by surgery followed by radiation therapy or concurrent chemoradiation therapy. Although new investigative techniques and improved radiation and surgical therapies have become available, survival after a HNC diagnosis has not improved substantially in the past decades [12]. This could be explained by most patients still being diagnosed with advanced stage HNC [7, 13, 14]. The 5‐year overall survival rate of patients diagnosed with HNC ranges from 20% to approximately 70% across different countries depending on the stage at diagnosis and the anatomical location of the tumour [15, 16]. A meta‐analysis of HNC survival in Africa included 24 studies published between 2002‐2022 and estimated a pooled overall 5‐year survival of 54% with a high between‐study heterogeneity [17]. Of note, only one study included in this meta‐analysis considered HIV status [18]. The Tanzania based study reported HIV to be significantly associated with poorer prognosis and HIV‐related complications to be one of the leading causes of mortality in HNC patients with HIV [18]. Only 10 of the 24 studies included in the meta‐analysis above were from sub‐Saharan Africa (Tanzania, Uganda, Sudan, Nigeria, South Africa and Cameroon) with an average 5‐year survival of approximately 30% [17]. Studies on the association of HIV status and HNC survival have found conflicting results. A study conducted in the United States (US) found HNC patients with HIV to have poorer 3‐year survival than those without HIV (49% vs 69%) [19]. In contrast, another US based study found that median survival among HNC patients with HIV was similar to the HNC survival in the general population (63 vs 61 months) but CD4 cell counts of <200 cells/μL were associated with poorer survival [20].

There is limited evidence on the impact of HIV infection on HNC survival in sub‐Saharan Africa where HNC survival is generally low, and the burden of HIV is high. To bridge this gap, we leveraged South African medical aid claims data to assess the association of HIV status and overall survival among insured HNC patients who underwent cancer treatment within six months of their diagnosis, and to examine factors associated with the type of treatment received.

## METHODS

### Data source

We sourced patient‐level data from a South African medical insurance scheme database which included both inpatient and outpatient reimbursement claims data, laboratory data, and demographics. We used data covering the period from 1 January 2011 to 1 July 2020. The claims data were coded using International Statistical Classification of Diseases and Related Health Problems (ICD‐10), International Classification of Diseases for Oncology (ICD‐O‐3), Current Procedural Terminology (CPT), Anatomical Therapeutic Chemical (ATC), National Pharmaceutical Product Interference (NAPPI) and National Reference Price List (NRPL) codes. The patient's vital status and date of death were obtained from the database and linkage with the South African National Population Register (NPR). The Human Research Ethics Committee of the University of Cape Town and the Ethics Committee of the Canton of Bern granted permission to analyse these data.

### Inclusion criteria and definitions

The analysis included individuals aged 18 years and above at the year of HNC diagnosis with at least two ICD‐10 codes for HNC located at one of five anatomical sites and having at least one cancer treatment code within 180 days of the HNC diagnosis. The HNC anatomical sites were: (1) mouth including the lip, tongue, gum, floor of mouth, palate and other parts of mouth (ICD‐10 codes C00‐C06), (2) salivary glands (C07‐C08), (3) pharynx including the tonsils, oropharynx, nasopharynx, pyriform fossa, hypopharynx and other mouth/pharynx (C09‐C14), (4) nasal including nose, sinuses and related structures (C30‐C31), and (5) larynx (C32). HIV status was determined based on the following indicators: ICD‐10 codes (B20‐B24, F02.4, O98.7, R75, Z21), laboratory data (positive HIV test or availability of HIV RNA viral load, CD4 cell count, or CD4 percentage measurements), ATC codes for antiretroviral therapy (ART), and identifiers linking patients to the Aid for AIDS (AfA), which is an HIV disease management programme. Individuals with two or more HIV indicators, with the first indicator recorded before or up to six months after HNC diagnosis were defined as having HIV. Individuals without any HIV indicators were considered HIV‐negative. We excluded individuals with only one HIV indicator, with a first HIV indicator recorded more than six months after HNC diagnosis, with missing information on age, without any follow‐up time after HNC diagnosis, and those who were not included in the linkage with the NPR. We defined a cancer as metastasized at the time of diagnosis if there was an ICD‐10 code for secondary malignancy (C77‐C79), or an ICD‐O‐3 morphology code for metastatic behaviour within 30 days of diagnosis. Otherwise, we defined the cancer as localized. We identified cancer treatments (radiotherapy, chemotherapy and surgery) using ICD‐10, CPT, NRPL and NAPPI codes (Table S1). We carried forward treatment codes recorded within 90 days before HNC diagnosis to the day of diagnosis to capture procedures that preceded the diagnosis.

### Statistical analysis

We compared patients' demographics and clinical characteristics stratified by HIV status and sex. We used separate logistic regression models to estimate odds ratios of receiving a specific type of cancer treatment (radiotherapy, chemotherapy or surgery) based on different patient characteristics. The multivariable logistic regression model included HIV status (positive, negative), age group (<40 years, 40–59 years, ≥60 years), sex (male, female), cancer anatomical site (mouth, salivary glands, pharynx, nasal, larynx, multiple sites), and cancer stage at diagnosis (metastasized, localized). We computed the overall survival of HNC patients using Kaplan–Meier curves, stratified by HIV status. We examined factors associated with all‐cause mortality using Cox proportional hazards models. The multivariable Cox model included HIV status, age group, sex, cancer anatomical site, cancer stage at diagnosis, and cancer treatment received. Time‐at‐risk started at the time of the HNC diagnosis and ended at a person's death, transfer from the medical aid scheme or database closure (1 July 2020), whichever came first. All statistical analyses were performed using R 4.4.1 (R Foundation for Statistical Computing, Vienna, Austria).

## RESULTS

### Study population

**FIGURE 1:**
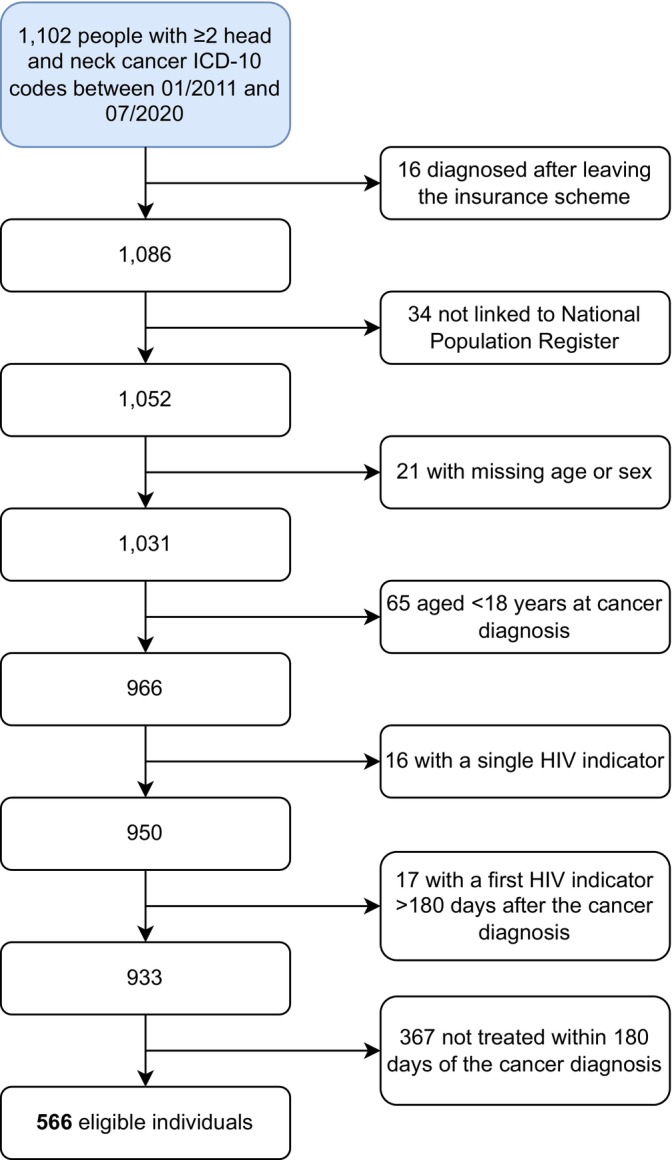
Flow diagram showing the number of included and excluded patients.

We included 566 people with HNC from the medical insurance database (January 2011 to July 2020), of whom 49 (9%) lived with HIV (Table 1, Figure 1). Patients with HIV were all on ART and were substantially younger at HNC diagnosis (median age: 52.5 years) than patients without HIV (median age: 61.6 years). Most patients were male (*n* = 383, 68%), and men tended to be older at HNC diagnosis (median age: 61.3 years) than women (median age: 59.3 years). Of 426 individuals with known information on population group, 193 were White (45.3%) and 188 were Black African (44.1%). Cancer of the mouth was the most common HNC site among patients with HIV (*n* = 14, 29%) and patients without HIV (*n* = 174, 34%). Salivary glands were the second most common HNC site in PWH, but only the fourth most common in people without HIV. A total of 94 individuals (17%) had HNC diagnoses at multiple sites. Cancer of the mouth was substantially more common among women than men (Table 2). About one‐third of HNC patients (*n* = 201, 35.5%) were diagnosed in the metastasized cancer stage.

**TABLE 1:** Characteristics and demographics at head and neck cancer diagnosis, stratified by HIV status.

Characteristics	Total (*N* = 566)	Without HIV (*N* = 517)	With HIV (*N* = 49)
Sex			
Male	383 (67.7)	349 (67.5)	34 (69.4)
Female	183 (32.3)	168 (32.5)	15 (30.6)
Median age at cancer diagnosis (IQR) [years]	60.6 (52.4, 69.2)	61.6 (54.1, 70.6)	52.5 (42.2, 57.3)
Age group at cancer diagnosis			
<40 years	49 (8.7)	39 (7.5)	10 (20.4)
40–59 years	222 (39.2)	189 (36.6)	33 (67.3)
≥60 years	295 (52.1)	289 (55.9)	6 (12.2)
Cancer site			
Mouth	188 (33.2)	174 (33.7)	14 (28.6)
Salivary glands	72 (12.7)	61 (11.8)	11 (22.4)
Pharynx	88 (15.5)	79 (15.3)	9 (18.4)
Nasal	31 (5.5)	28 (5.4)	3 (6.1)
Larynx	93 (16.4)	88 (17.0)	5 (10.2)
Multiple sites	94 (16.6)	87 (16.8)	7 (14.3)
Cancer stage at diagnosis			
Localized	365 (64.5)	332 (64.2)	33 (67.3)
Metastasized	201 (35.5)	185 (35.8)	16 (32.7)
Cancer treatment received			
Radiation only	16 (2.8)	15 (2.9)	1 (2.0)
Chemotherapy only	87 (15.4)	85 (16.4)	2 (4.1)
Surgery only	98 (17.3)	83 (16.1)	15 (30.6)
Combined treatments	365 (64.5)	334 (64.6)	31 (63.3)

*Note*: Results are reported as numbers and percentages if not otherwise stated.

**TABLE 2:** Characteristics and demographics at head and neck cancer diagnosis, stratified by sex.

Characteristics	Male (*N* = 383)	Female (*N* = 183)
HIV status		
Positive	34 (8.9)	15 (8.2)
Negative	349 (91.1)	168 (91.8)
Median age at cancer diagnosis (IQR) [years]	61.3 (54.6, 68.9)	59.3 (46.9, 70.2)
Age group at cancer diagnosis		
<40 years	20 (5.2)	29 (15.8)
40–59 years	153 (39.9)	69 (37.7)
≥60 years	210 (54.8)	85 (46.4)
Cancer stage at diagnosis		
Localized	246 (64.2)	119 (65.0)
Metastasized	137 (35.8)	64 (35.0)
Cancer treatment received		
Radiation only	11 (2.9)	5 (2.7)
Chemotherapy only	54 (14.1)	33 (18.0)
Surgery only	55 (14.4)	43 (23.5)
Combined treatments	263 (68.7)	102 (55.7)
Cancer site		
Mouth	108 (28.2)	80 (43.7)
Salivary glands	44 (11.5)	28 (15.3)
Pharynx	62 (16.2)	26 (14.2)
Nasal	21 (5.5)	10 (5.5)
Larynx	84 (21.9)	9 (4.9)
Multiple sites	64 (16.7)	30 (16.4)

*Note*: Results are reported as numbers and percentages if not otherwise stated.

### Treatment

More than three quarters (*n* = 435, 77%) of the study population were treated with chemotherapy within 180 days of their HNC diagnosis while surgery (*n* = 328, 58%) and radiotherapy (*n* = 323, 57%) were less common. We found no clear association between a person's HIV status and the type of treatment they received in unadjusted (Table S2) and adjusted analyses (Table 3). However, women were less likely to receive radiotherapy than men (adjusted odds ratio [aOR] 0.57; 95% confidence interval [CI] 0.39–0.84). Patients with metastasized HNC were more than twice as likely to be treated with chemotherapy (aOR 2.46; 95% CI 1.54–4.05) than patients with localized tumours. The pharyngeal (aOR 0.24; 95% CI 0.14–0.41) and nasal cancers (aOR 0.17; 95% CI 0.07–0.39) were substantially less likely to be operated on than mouth cancers. In contrast, most cancer sites apart from salivary glands were more likely to be treated with chemotherapy or radiation therapy, compared with mouth cancers (Table 3).

**TABLE 3:** Adjusted odds ratios for receiving a specific cancer treatment within 6 months after a head and neck cancer diagnosis.

Characteristic	Patients treated (*N* = 323)	aOR for radiotherapy (95% CI)	Patients treated (*N* = 435)	aOR for chemotherapy (95% CI)	Patients treated (*N* = 328)	aOR for surgery (95% CI)
HIV status						
Negative	293	1	402	1	297	1
Positive	30	1.16 (0.61–2.24)	33	0.63 (0.31–1.28)	31	1.45 (0.75–2.87)
Sex						
Male	238	1	303	1	217	1
Female	85	0.57 (0.39–0.84)	132	0.72 (0.46–1.14)	111	1.14 (0.77–1.69)
Age category						
<40 years	26	1.04 (0.54–2.02)	38	1.06 (0.49–2.41)	25	0.68 (0.35–1.32)
40–59 years	133	1.20 (0.82–1.75)	165	0.81 (0.52–1.27)	127	0.94 (0.64–1.38)
≥60 years	164	1	232	1	176	1
Cancer site						
Mouth	85	1	133	1	127	1
Salivary glands	37	1.39 (0.79–2.45)	47	0.91 (0.50–1.68)	50	1.12 (0.62–2.07)
Pharynx	57	2.05 (1.20–3.53)	81	4.80 (2.18–12.18)	29	0.24 (0.14–0.41)
Nasal	21	2.66 (1.19–6.31)	27	3.00 (1.09–10.61)	8	0.17 (0.07–0.39)
Larynx	56	1.82 (1.07–3.12)	70	1.37 (0.76–2.54)	54	0.70 (0.41–1.20)
Multiple sites	67	2.96 (1.73–5.15)	77	1.88 (1.02–3.59))	60	0.86 (0.51–1.45)
Cancer stage at diagnosis						
Localized	190	1	261	1	209	1
Metastasized	133	1.91 (1.31–2.79)	174	2.46 (1.54–4.05)	119	1.15 (0.79–1.67)

Abbreviations: aOR, adjusted odds ratio; CI, confidence interval.

### Survival

**FIGURE 2:**
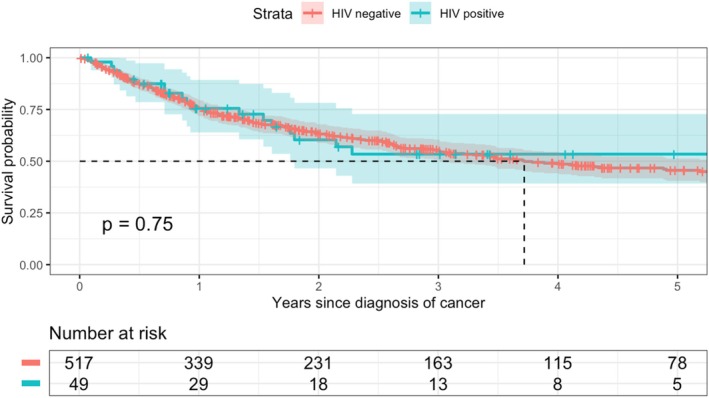
Kaplan–Meier overall survival curves, stratified by HIV status.

Overall, 241 patients died during the follow‐up period. The median survival was 3.78 years (95% CI 3.01–6.08), and the five‐year survival was 46.0% (95% CI 41.2%–51.5%). The Kaplan–Meier analysis (Figure 2) found comparable 1‐year survival estimates for HNC patients with HIV (75.5%; 95% CI 63.9%–89.3%), and those without HIV (75.5%; 95% CI 71.8%–79.5%). Similarly, the estimated 5‐year survival was 53.4% (95% CI 39.2%–72.8%) among HNC patients with HIV and 45.6% (95% CI 40.5%–51.3%) among those without HIV. The results from the Cox regression analysis of factors associated with time to death are shown in Table 4. The unadjusted analysis suggested that HIV status was not associated with mortality (hazard ratio [HR] 0.93; 95% CI 0.57–1.50). However, after adjusting the analysis for age group, sex, cancer anatomical site, cancer stage at presentation, and cancer treatment received, the risk of death was higher among patients with HIV compared with those without HIV (adjusted HR [aHR] 1.68; 95% CI 1.00–2.81). Female HNC patients had a lower risk of death than male HNC patients in the unadjusted analysis (HR 0.69; 95% CI 0.53–0.92), but this association was attenuated in the adjusted analysis (aHR 0.81; 95% CI 0.61–1.09). We found no clear association in the adjusted analysis between the risk of death for patients with other HNCs compared to mouth cancers, although those with salivary gland cancers (aHR 0.66; 95% CI 0.40–1.09) or larynx cancer (aHR 0.67; 95% CI 0.44–1.02) tended to have lower mortality rates. Compared with patients receiving combined cancer treatments, those undergoing radiation monotherapy had higher mortality rates, whereas patients undergoing surgery monotherapy had lower mortality rates (Table 4).

**TABLE 4:** Unadjusted and adjusted hazard ratios for all‐cause mortality after a head and neck cancer diagnosis.

Characteristic	Deaths (*N*)	Unadjusted hazard ratio (95% CI)	Adjusted hazard ratio (95% CI)
HIV status			
Negative	223	1	1
Positive	18	0.93 (0.57–1.50)	1.68 (1.00–2.81)
Sex			
Male	172	1	1
Female	69	0.69 (0.53–0.92)	0.81 (0.61–1.09)
Age category			
<40 years	11	0.40 (0.22–0.74)	0.31 (0.16–0.59)
40–59 years	89	0.78 (0.60–1.01)	0.79 (0.60–1.04)
≥60 years	141	1	1
Cancer site			
Mouth	78	1	1
Salivary glands	20	0.61 (0.37–1.00)	0.66 (0.40–1.09)
Pharynx	42	1.27 (0.87–1.85)	1.02 (0.70–1.51)
Nasal	19	1.69 (1.02–2.79)	1.28 (0.77–2.14)
Larynx	33	0.83 (0.55–1.25)	0.67 (0.44–1.02)
Multiple sites	49	1.18 (0.83–1.69)	1.03 (0.72–1.48)
Cancer stage at diagnosis			
Localized	145	1	1
Metastasized	96	1.38 (1.07–1.79)	1.08 (0.82–1.42)
Cancer treatment received			
Combined treatments	170	1	1
Chemotherapy only	46	1.19 (0.86–1.65)	1.33 (0.95–1.88)
Surgery only	15	0.23 (0.13–0.38)	0.23 (0.13–0.40)
Radiation only	10	1.70 (0.90–3.22)	2.23 (1.15–4.30)

Abbreviation: CI, confidence interval.

## DISCUSSION

Two‐thirds of the HNC patients included in our analysis were male and 9% lived with HIV. Patients with HIV were substantially younger at HNC diagnosis than those without HIV, but the sex and cancer stage distribution was similar in the two groups. We found no clear association between HIV status and type of cancer treatment received. However, all‐cause mortality was 68% higher among patients with HIV than among those without HIV.

Our study is one of the few in sub‐Saharan Africa that assessed HNC survival by HIV status and derives its strength from the large sample size, compared to other studies in the African region [21, 22], and the linkage with the NPR to validate the vital status information. However, several limitations need to be considered. The included number of HNC patients with HIV was relatively low and thus results for this patient group, including the distribution of the cancer site, should be interpreted cautiously. The use of medical claims data can lead to misclassification. We mitigated the risk of including false‐positive HNC diagnoses by only including patients with at least two recorded ICD‐10 codes for HNC, supplemented by cancer treatment codes. By restricting our analysis to treated HNC patients, however, we limited the generalizability of our results. Similarly, we required patients with HIV to have at least two HIV indicators and assumed that the absence of HIV indicators indicated that patients were HIV negative. This may have led to an underestimation of the HIV prevalence in our study population. Nevertheless, as we used a wide range of indicators for HIV including laboratory tests, ICD‐10 codes, and ART records, we expect this misclassification to be minimal. The use of ICD‐10 codes for metastatic cancer is an imperfect proxy for cancer stage, and thus, our results may not be directly comparable to studies that used clinical staging information. Furthermore, we did not have information on the HPV status of the HNCs included in our analysis and could not assess the impact of HPV status on treatment and survival. We also did not have precise information on the cause of death and, therefore, focused our analysis on all‐cause mortality. Lastly, the study population only included people with medical insurance who tend to have lower HIV prevalence than the general South African population, and better access to cancer diagnostics and treatment. Therefore, our results are unlikely to be generalizable to the general South African population.

The male predominance of HNC is well established together with its risk factors [2]. This could be due to sex differences in behavioral factors such as smoking and alcohol consumption [23, 24], occupational exposures, and sex‐specific hormonal and immunological differences [25]. In our study, two‐thirds of the HNC patients were male with no difference in the sex distribution between HNC patients with and without HIV. Another South African study, however, found a higher proportion of women among HNC patients with HIV than those without HIV [26], reflecting the higher HIV prevalence among women than men in South Africa. This pattern is partly driven by socio‐economic disparities and might, thus, be less pronounced in our more affluent study population. Moreover, differences in the definition of HNCs between the two studies may also contribute to the observed variability. In line with other studies [27, 28], we found that HNC patients with HIV were substantially younger at cancer diagnosis than patients without HIV, likely reflecting the impact of immune dysfunction. HNC is often diagnosed late, with more than 50% of HNC patients presenting with locally advanced cancer or distant metastases [7, 29, 30, 31]. In our study, about one‐third of patients had metastasized cancer at diagnosis. It is possible that our insured study population was more often diagnosed in early stages of HNC cancer because individuals with medical insurance tend to have access to improved HNC diagnostics and better health care services [32, 33]. However, estimates across studies also need to be compared cautiously due to differences in staging definition. We relied on ICD‐10 codes for distant metastasis for staging while other studies used clinical staging and included both locally advanced (stage III) and metastatic cancer (stage IV) in their definition of advanced cancer. We and others [2, 26, 28, 34] found that, the mouth was the most common HNC site, possibly due to frequent exposure of the mouth to carcinogens such as tobacco and alcohol [7]. In contrast, North Africa and Southeast Asia report nasopharynx as the most common HNC site, which has been linked to EBV infection, genetic predisposition, and environmental exposures [8].

Treatment of HNCs is challenging because of the large variation in tumour sites and histological subtypes [35, 36]. It typically involves surgery, radiation therapy, chemotherapy, or a combination thereof. Optimal treatment planning must consider tumor site and stage, patient characteristics and preferences, and available resources and expertise. A systematic review found a combination of radiotherapy and chemotherapy to be the most common treatment modalities for HNC in Africa [17]. The scarcity of trained head and neck surgeons — only 19 across Africa in 2021 — may partly explain this finding [17]. In our study, most patients underwent a combination of treatments. The type of treatment received was not clearly associated with HIV status, which is in line with other findings from the African region [21]. Of note, all HNC patients with HIV in our analysis received ART.

The overall survival of HNC has not improved substantially over the past decades, despite advances in diagnostics and treatment modalities [12]. The overall 5‐year survival in our study was 46%, which is higher than the approximately 30% found in a study done among HNC patients treated with curative or palliative intent at Groote Schur Hospital in Cape Town, South Africa [34]. However, it is lower than the pooled estimate of 54% reported by a meta‐analysis of African studies. Notably, between‐study heterogeneity in this meta‐analysis was large with 5‐year survival estimates varying greatly between individual studies [17]. In contrast, 5‐year HNC survival in high‐income countries like the US typically reaches more than 90% [37]. This geographic variation in HNC survival is likely explained by socioeconomic disparities and differences in access to health care [38]. In our Kaplan Meier analysis, without accounting for other factors, survival did not differ by HIV status. Similar findings were reported for Botswana where HNC patients with HIV had similar survival to patients without HIV [21]. However, the association between HIV status and HNC survival was confounded by age, as HNC patients with HIV were younger than those without HIV, and younger patients have better overall survival in general. Additionally, more HNC patients with HIV underwent surgery only which was associated with a better survival than other types of treatment. In our adjusted analysis, HNC patients with HIV had a 68% higher risk of death from any cause than patients without HIV. Elevated all‐cause mortality among HNC patients with HIV has also been observed in other settings, with estimates indicating a 98% increase in the US and a 14% increase in Botswana [19, 21]. We were unable to estimate cancer‐specific mortality but all HNC patients with HIV in our study received ART and, therefore, competing HIV‐related mortality is expected to be low. Further research is needed to better understand reasons for these disparities in survival outcomes between HNC patients with and without HIV including potential differences in tumour biology, treatment‐related toxicities, or treatment protocols.

## CONCLUSION

We found a low overall 5‐year survival of less than 50% among medically insured HNC patients in South Africa. This highlights the pivotal role of early detection and treatment to improve HNC survival. Furthermore, the risk of death among insured HNC patients with HIV was higher than among patients without HIV, underscoring the importance of tailored cancer care strategies for HNC patients with HIV.

## AUTHOR CONTRIBUTIONS

PKM and ER conceptualized the study; ADH and ER were involved in the funding acquisition; NF provided resources; YR, ADH and CC were involved in the data management; PKM performed the data analysis with support from YR; PKM and ER wrote the first draft of the manuscript; all authors contributed to the interpretation of the results, reviewed the manuscript, and agreed with the final version.

## FUNDING INFORMATION

This work was supported by the US National Institutes of Health's National Institute of Allergy and Infectious Diseases; the Eunice Kennedy Shriver National Institute of Child Health and Human Development; the National Cancer Institute; the National Institute of Mental Health; the National Institute on Drug Abuse; the National Heart, Lung, and Blood Institute; the National Institute on Alcohol Abuse and Alcoholism; the National Institute of Diabetes and Digestive and Kidney Diseases; and the Fogarty International Center (Grant Number U01AI069924). The Swiss National Science Foundation (SNSF) supported ADH (Grant Number 193381). The funder did not play a role in the design of the study; the collection, analysis and interpretation of the data; the writing of the manuscript; and the decision to submit the manuscript for publication.

## CONFLICT OF INTEREST STATEMENT

The authors have no conflicts of interest to declare.

## PATIENT CONSENT STATEMENT

Individual patient consent was not obtained for this analysis, as the study used de‐identified, routinely collected data. The study was approved by the relevant institutional ethics committees, which granted a waiver of informed consent in accordance with local regulations and ethical guidelines.

## Supporting information


**Supplementary Table S1:** Reimbursement claims codes used for definition of variables and patient selection.
**Supplementary Table S2:** Unadjusted odds ratios (OR) and 95% confidence intervals (CI) for receiving a specific cancer treatment within 6 months after a head and neck cancer diagnosis.

## Data Availability

Data were obtained from the International Epidemiology Databases to Evaluate AIDS–Southern Africa (IeDEA‐SA) and for inquiries about the data, readers can contact them through the online form available at https://www.iedea-sa.org/contact-us/. Further information is available from the corresponding author upon request.
