# Supplementary material for: Survival among medically insured and treated head and neck cancer patients with and without HIV in South Africa
Source: HIV Med. 2025 Nov 19;26(12):1973–82. doi: 10.1111/hiv.70143 (PMC12666250; doi:10.1111/hiv.70143)
Supplement: Supplementary file 1 — Supplementary Table S1: Reimbursement claims codes used for definition of variables and patient selection. Supplementary Table S2: Unadjusted odds ratios (OR) and 95% confidence intervals (CI) for receiving a specific cancer treatment within 6 months after a head and neck cancer diagnosis. [file HIV-26-1973-s001.docx]

**Supplementary Materials**

**Survival Among Treated Head and Neck Cancer Patients With and Without HIV in South Africa**

**Supplementary Table S1:** Reimbursement claims codes used for definition of variables and patient selection**.**

**Supplementary Table 2:** Unadjusted odds ratios (OR) and 95% confidence intervals (CI) for receiving a specific cancer treatment within 6 months after a head and neck cancer diagnosis

**Supplementary Table S1:** Reimbursement claims codes used for definition of variables and patient selection**.**

| **Definition** | **Code Type** | **Codes** |
| --- | --- | --- |
| **Head and Neck Cancer** | ICD-10 | C00-C14, C30-32 |
| **HIV** | ICD-10 | B20-B24, F02.4, O98.7, R75, Z21 |
| **ART** | ATC | J05AE, J05AF, J05AG, J05AJ, J05AR |
| **Cancer stage** | ICD-10 | C77-C79.9 |
|  | ICD-O-3 | Morphology behaviour code /6 |
| **Radiotherapy** | ICD-10 | Z51.0 |
|  | NRPL | 5635-5861, 5882-5893, 88801-88819 |
|  | CPT | 57155, 57156, 58346, 77014, 77295-77620, 77750-77799, 79005, 79101, 79403 |
|  | NAPPI | 00310, 907-909, |
| **Chemotherapy** | ICD-10 | Z51.1, Z51.2 |
|  | CPT | 96413-96416 |
|  | NRPL | 5790-5795, 88851, 88853-88855, 99951, 99953-99955, 99959 |
|  | ATC | L01AA01, L01BA01, L01BL02, L01BL05, L01BL06, L01CA04, L01CB01, L01CD01, L01CD02, L01CD03, L01DB01, L01DB03, L01DC01, L01EB03, L01EX08, L01FE01, L01FF01, L01FF02, L01FF07, L01FF13, L01XA01-02, L01XX05 |
|  |  |  |
|  |  |  |
|  |  |  |
|  | NAPPI | 723266018, 723274002,723282005, 723282014, 72330400, 723304017, 713873,300488,715323,3004230,3002821,715252,815802,717964,718304,3003452,3004887,716717,713874,3004890,715322,3004229,3002820,715251,815799,717963,718303,3003442,716719,723214,3001285,722783,3003052,722962,870072,3006804,723225,3001286,722784,3006805,3003053,722963,870080,3003306,3001749,3004004,7193893002451,723793,3003556,3003459,814768,723381,719959,715749,3003307,3001752,3004003,719390,3002452,723794,3003557,3003460,814741,723387,719960,715807,721804,713679,3004245,823635,710404,721800,721804,3005755,718301,3007786,837008,721802,894042,867411,719985,720412,3004522,894044,721805,713680,3004247,823643,710405,721801,3005759,899468,3007787,718302,711367,720402,3004239,825069,711368,720403,3004240,704097,840874,3004242,707364,840866,720381,720382,840858,825573,825581,782505,3007785,840831,782513,716228,714218,714241,716240,710406,716239,714219,714220,716241,710408,719861,850926,3005394,710402,841021,720616,712753,716632,716179,715014,716950,717009,714757,841730,700431,3006704,3005832,716909,720617,712754,716633,715016,717011,714758,704126,700432,3006705,716180,716910,720614,704208,716631,703386,716178,715013,717008,714756,799777,700427,711892,3006703,716908,3005833,712613,715822,822655,716039,715784,721304,716184,700191,3003765,700192,3003766,715821,819263,721305,712611,716040,715783,716185,711366,782491,711136,726877,712504,742465,782556,825557,703658,704145,3002828,3002827,721059,3000046,3000047,3000048,715052,710034,723729,3000113,3000114,3005203,721586,721585,3005202, |
| **Surgery** | CPT | 11621,15731,15732,15756,15757,20969,21015,21016,21025,21044,21045,21554,21556,21557,21558,31225,31230,31300,31360,31365,31367,31368,31370,31375,31380,31382,31390,31395,38700,38720,38724,40810,40812,40814,40816,40819,40820,40842,41100,41105,41108,41110,41112,41113,41114,41116,41120,41130,41135,41140,41145,41150,41153,41155,41530,41820,41825,41826,41827,41850,42100,42104,42106,42107,42120, 42140,42160,42410,42415,42420,42425,42426,42440,42450,42808,42821,42826,42842,42844,42845,42860,42870,42890,4289242894,42999 |
|  | NRPL | 1101,1102,1105,1119,1125,1445,1463,1465,1469,1471,1473,1487,1501,1503,1504,1505,1507,1509,1511,1515,1517,1519,1521,1523,1525,1526,1527,1529,4914,8973,8975,8977,9019,9069,9095 |

**Supplementary Table 2:** Unadjusted odds ratios (OR) and 95% confidence intervals (CI) for receiving a specific cancer treatment within 6 months after a head and neck cancer diagnosis.

| **Characteristic** | **OR for radiotherapy (95% CI)** | **OR for chemotherapy (95% CI)** | **OR for surgery**  **(95% CI)** |
| --- | --- | --- | --- |
| **HIV status** |  |  |  |
| Negative | 1 | 1 | 1 |
| Positive | 1.21 (0.67-2.23) | 0.59 (0.32- 1.14) | 1.28 (0.70-2.38) |
| **Sex** |  |  |  |
| Male | 1 | 1 | 1 |
| Female | 0.53 (0.37-0.75) | 0.68 (0.46- 1.03) | 1.18 (0.82-1.69) |
| **Age category** |  |  |  |
| <40 years | 0.90 (0.49-1.66) | 0.94 (0.47- 2.02) | 0.70 (0.38-1.30) |
| 40-59 years | 1.19 (0.84-1.70) | 0.79 (0.52- 1.19) | 0.90 (0.63-1.29) |
| ≥60 years | 1 | 1 | 1 |
| **Cancer site** |  |  |  |
| Mouth | 1 | 1 | 1 |
| Salivary glands | 1.28 (0.74-2.21) | 0.78 (0.44- 1.40) | 1.09 (0.61-1.99) |
| Pharynx | 2.23 (1.33-3.79) | 4.79 (2.21-11.98) | 0.24 (0.14-0.40) |
| Nasal | 2.54 (1.16-5.92) | 2.79 (1.03- 9.77) | 0.17 (0.07-0.38) |
| Larynx | 1.83 (1.11-3.06) | 1.26 (0.72- 2.25) | 0.67 (0.40-1.11) |
| Multiple sites | 3.01 (1.78-5.18) | 1.87 (1.03- 3.54) | 0.85 (0.51-1.43) |
| **Cancer stage at diagnosis** |  |  |  |
| Localized | 1 | 1 | 1 |
| Metastasized | 1.80 (1.26-2.58) | 2.57 (1.63- 4.15) | 1.08 (0.76-1.54) |
